# Kinetic Studies to Elucidate Impaired Metabolism of Triglyceride-rich Lipoproteins in Humans

**DOI:** 10.3389/fphys.2015.00342

**Published:** 2015-11-20

**Authors:** Martin Adiels, Adil Mardinoglu, Marja-Riitta Taskinen, Jan Borén

**Affiliations:** ^1^Department of Molecular and Clinical Medicine/Wallenberg Laboratory, University of GothenburgGothenburg, Sweden; ^2^Health Metrics Unit, Sahlgrenska Academy, University of GothenburgGothenburg, Sweden; ^3^Department of Biology and Biological Engineering, Chalmers University of TechnologyGothenburg, Sweden; ^4^Science for Life Laboratory, KTH - Royal Institute of TechnologyStockholm, Sweden; ^5^Heart and Lung Centre, Helsinki University Hospital and Research Programs' Unit, Diabetes & Obesity, University of HelsinkiHelsinki, Finland

**Keywords:** very low density lipoproteins, apoB, multocomnpartmental modeling, kinetics, stable isotopes

## Abstract

To develop novel strategies for prevention and treatment of dyslipidemia, it is essential to understand the pathophysiology of dyslipoproteinemia in humans. Lipoprotein metabolism is a complex system in which abnormal concentrations of various lipoprotein particles can result from alterations in their rates of production, conversion, and/or catabolism. Traditional methods that measure plasma lipoprotein concentrations only provide static estimates of lipoprotein metabolism and hence limited mechanistic information. By contrast, the use of tracers labeled with stable isotopes and mathematical modeling, provides us with a powerful tool for probing lipid and lipoprotein kinetics *in vivo* and furthering our understanding of the pathogenesis of dyslipoproteinemia.

## Introduction

The most abundant lipids in plasma are: triglycerides, cholesterol, cholesterol esters, and phospholipids. Since lipids are water-insoluble they have to be transported in lipoprotein particles. They consist of a hydrophobic core of triglycerides and cholesterol esters, shielded from the water by a surface monolayer of phospholipids, unesterified cholesterol, and specific proteins (Mahley et al., 1984). The protein components of the lipoprotein are known as apolipoproteins (apo) (Mahley et al., 1984). The amount of lipids and proteins in the lipoprotein particles affect their density—the lower the density of a lipoprotein, the more lipids it contains relative to protein. Depending on function and hydrated density, the lipoproteins are traditionally divided into four major classes. These are chylomicrons, very low-density lipoproteins (VLDL), low-density lipoproteins (LDL), and high-density lipoprotein (HDL).

Chylomicrons and VLDL particles are the major carriers of triglycerides in the circulation. Chylomicrons are synthesized in the intestine and carry dietary lipids absorbed by the intestine. VLDL particles are synthesized by the liver. The function of these lipoprotein particles is to transport and deliver triglycerides to adipose tissue and muscles. Elevated triglycerides in plasma are associated with increased risk for cardiovascular disease (CVD).

In order to prevent and treat disturbances in metabolism of triglyceride-rich lipoproteins, it is necessary to clarify the underlying mechanism(s). The hypertriglyceridemia can either be caused by increased secretion, conversion, or catabolism of lipoprotein particles of triglyceride-rich lipoproteins. Although static s measurements of plasma lipids and functional assays may give some information, in the end, it is necessary to study the true unit of function (the integrated metabolic pathway) to understand the complexity of lipoprotein metabolism (Chan et al., 2004a,b; Adiels et al., 2008). Therefore, kinetic studies with stable isotopes are critical to explore and clarify the pathophysiology of lipid disorders in humans (Chan et al., 2004a,b; Adiels et al., 2008). The aim of this review is to illustrate how kinetic studies have furthered our understanding of impaired human lipoprotein metabolism.

## Metabolism of apoB-containing lipoproteins

**Figure 1:**
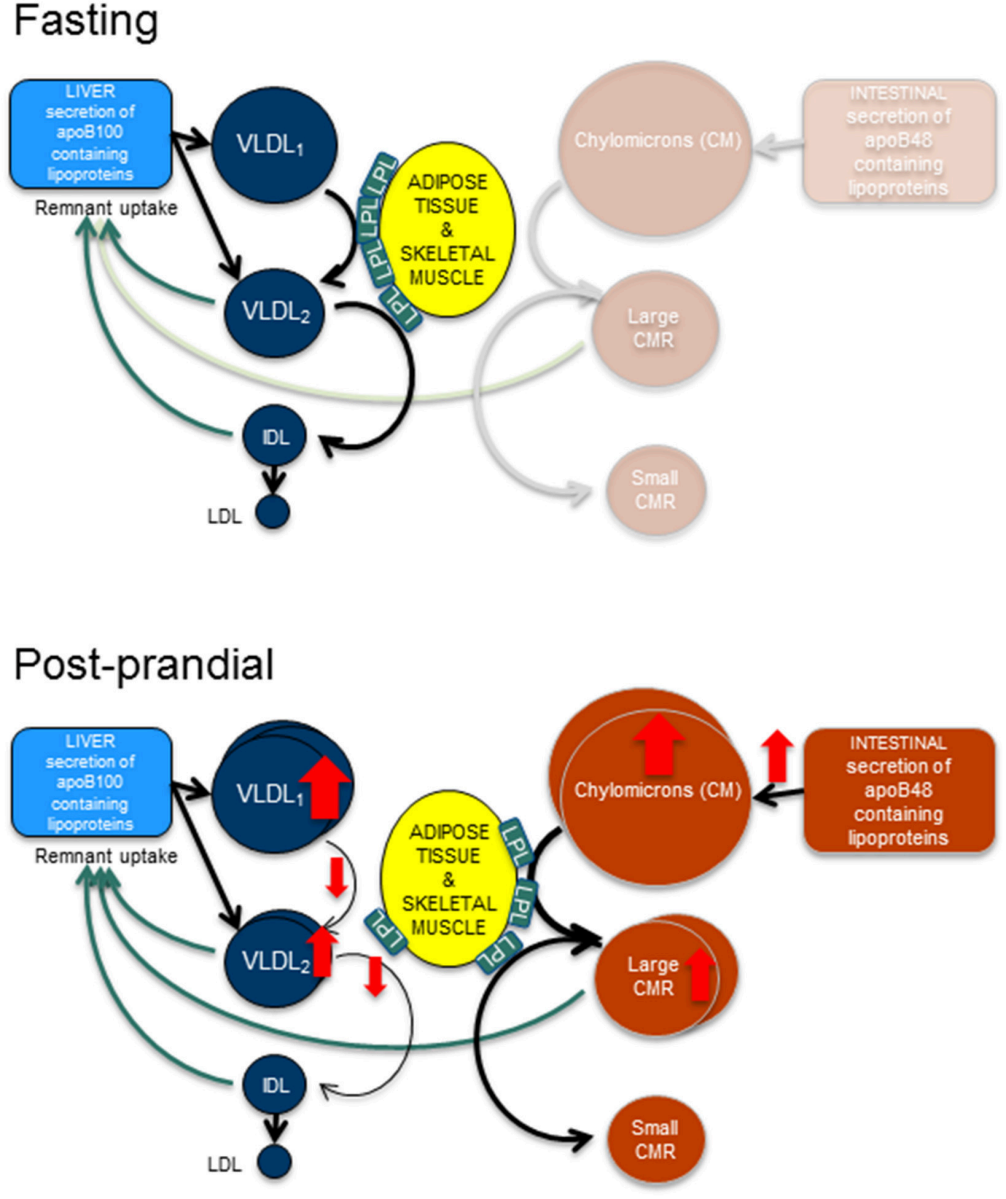
**Fasting and post-prandial triglyceride metabolism**. In fasting, apoB100 containing lipoproteins are secreted from the liver and are hydrolyzed by lipoprotein lipase (LpL) to form smaller and denser particles. Hence, very low density lipoprotein (VLDL)_1_ particles become VLDL_2_ particles and VLDL_2_ particles become intermediate density lipoproteins (IDL). After remodeling with cholesterol ester transfer protein and hepatic lipase the end product are the low density lipoproteins (LDL). In the post-prandial phase, the intestine secretes triglyceride rich apoB48 containing lipoproteins, the chylomicrons. After hydrolyzation by LpL, large and small chylomicron remnants (CMR) are formed. This process likely occupies the majority of available LpL, thus resulting in slower turn-over of the apoB100 containing lipoproteins and causing an accumulation of these lipoproteins.

Triglyceride-rich lipoproteins in the circulation are a mixture of chylomicrons (synthesized in the intestine) and VLDL particles (synthesized in the liver) (Figure 1). Each of these lipoproteins contains one molecule of apolipoprotein B (apoB). apoB is a large hydrophobic protein that remains bound to the lipoprotein particles (Segrest et al., 2001). This unique characteristic of apoB makes it possible to use apoB as a tool to trace the intravascular kinetics of the triglyceride-rich lipoproteins.

apoB is present in two different lengths; apoB100 and apoB48. The shorter form apoB48 corresponds to the amino-terminal 48% of apoB100. It is in humans exclusively synthesized in the intestine, and thus present on intestinal-derived chylomicrons and their remnants. The longer form, apoB100 is synthesized in the liver and present on VLDL, IDL, and LDL. Both apoB100 and apoB48 are coded by the same gene, but the shorter apoB48 is generated as a result of a posttranscriptional process called “RNA editing” that converts a cytidine-to-uridine (C-to-U) that generates a stop-codon and thus a truncated form of the full-length protein. In humans, this posttranscriptional RNA editing occurs in the intestine only but in certain animals such as rodents and dogs, the process occurs also in the liver (Powell et al., 1987).

Why is apoB48 synthesized by this complex mechanism in the Intestine? The explanation is still unclear but it has been shown that apoB48-containing lipoproteins can carry more lipids than apoB100-containing lipoproteins. This is important since the chylomicrons must have capacity to rapidly and efficiently absorb large amounts of dietary lipids and a meal (Hussain, 2014). Once synthesized, chylomicrons are secreted into the lymphatic vessels until they enter into the bloodstream close to the heart. Thus, they are delivered directly to adipose tissue and muscles without first being metabolized by the liver. In the circulation, chylomicron-triglycerides are hydrolyzed by the enzyme lipoprotein lipase, which is present on the endothelial cells in the heart, muscle, and adipose tissue. The released free fatty acids are then taken up these tissues where they are stored or used for energy production. The regulation is LPL is controlled transcriptionally and post-transcriptionally. On the endothelial cells, LPL is activated by apoC-II and inhibited by apoC-III and ANGPTL4.

When the triglycerides are removed from the hydrophobic core of the chylomicrons, they shrink in size and become chylomicron remnants. The smaller chylomicron remnants are cleared from the circulation by the liver (Mahley and Ji, 1999; MacArthur et al., 2007; Williams, 2008; Williams and Chen, 2010). Recent studies have shown that chylomicron remnants are atherogenic and directly involved in atherogenesis. The explanation for this is that chylomicrons in addition to triglycerides also contain some cholesterol esters. When triglycerides are removed from the lipoprotein particle, the cholesterol esters remain. Thus, the chylomicron remnants become enriched in cholesteryl esters.

The liver secretes apoB100-containing VLDL particles. In fact, the liver produces two different forms of VLDL; larger VLDL_1_ and smaller VLDL_2_ particles. The triglyceride-rich VLDL_1_ particles carry most of the plasma triglycerides and have been shown to be the major determinant for the variation of plasma triglycerides in both healthy subjects and individuals with type 2 diabetes (Hiukka et al., 2005; Boren et al., 2014; Taskinen and Boren, 2015). The formation of VLDL_1_ is highly associated with fatty liver (Adiels et al., 2008; Boren et al., 2013). Indeed, subjects with non-alcoholic fatty live disease (NAFLD) have increased VLDL_1_ production (Adiels et al., 2006b).

VLDL-triglycerides are hydrolyzed by the same LPL-dependent mechanisms as chylomicrons, Figure 1. As VLDL-triglycerides are hydrolyzed, the density of the lipoprotein increases and they become intermediate density lipoproteins (IDL) and subsequently LDL. Like chylomicrons, also VLDL contains mainly triglycerides but also some cholesterol esters that become enriched in the lipoprotein particle when the triglycerides are removed. Thus, the end-product of the lipolytic cascade, LDL contains mainly cholesterol esters and is the major determinant of cholesterol in plasma. LDL particles are removed from the circulation by the LDL-receptor on the hepatocytes. The importance of the LDL-receptor for clearance of LDL particles is illustrated by the genetic disorder, familial hypercholesterolemia caused by mutation in the LDL-receptor. The disease is associated with hypercholesterolemia and premature CVD.

Patients with obesity and insulin resistance have a characteristic atherogenic dyslipidemia characterized by increased plasma triglycerides, excessive postprandial lipemia (i.e., rise in triglyceride-rich lipoproteins after eating) postprandial hyperlipidemia, and low concentrations of HDL cholesterol (Taskinen, 2003; Adiels et al., 2006a). Interestingly, these lipid disturbances are not isolated abnormalities but metabolically linked to each other (Taskinen, 2005), and they appear years before type 2 diabetes is diagnosed (Taskinen, 2003).

## Principles of tracer methodology

To simplest approach to study the kinetics of a molecule (i.e., the tracee) is to introduce the same molecule (tracer), but labeled into the system (Chan et al., 2004a,b). This process has many advantages but also obvious concerns, especially in human studies. The alternative is to introduce a labeled precursor of the molecule of interest. Ideally, the tracer should be easily detected and quantified, and not affect the system. Usually, kinetic studies are performed in a steady-state, where the rates of input and output for a given unlabeled tracee substance are equal and time-invariant. Thus, the information provided by the tracer reflects the behavior of the tracee (Barrett et al., 2006). At various times, the amount of tracer is quantified to provide a kinetic curve. Then a mathematical model is constructed to extract all of the information contained in the kinetic curve. By fitting a model to the data, it is possible to calculate the parameters of the model that characterize the flux of molecules between kinetically homogeneous pools of molecules. Historically, the radioactive isotopes were used as tracers, but today naturally occurring non-radioactive stable isotopes are almost exclusively used in human studies. The technical advances in mass-spectrometry technologies now permit accurate measurement of stable isotopes in smaller samples and in low concentrations (Barrett et al., 2006; Adiels et al., 2010).

A tracer can be introduced into the system either as a bolus injection or as a constant infusion given immediately after a priming dose. The bolus administration of the tracer is suitable to study kinetics of molecules with a relatively slow rate of turnover since the enrichment curves (the tracer/tracee ratios) after a bolus injection corresponds to the impulse response of the system. Furthermore, it also enables determination of newly synthesized particles, since the intracellular precursor enrichment is greater at the start of the study. Primed constant infusions require a longer time to achieve a plateau of isotopic enrichment but can be appropriate for kinetic studies of molecules with rapid turnover.

## Modeling of lipoprotein metbolism

Mathematical modeling enables us to better understand experimental observations. Predictions derived from model predictions can subsequently be experimentally tested. Toward this goal, known pathways are described by a set of differential equations, thereby allowing quantitative estimates to be derived. In multi-compartment models, molecules of interest move among different compartments of a system. Each compartment is assumed to be a homogeneous entity within which the entities being modeled are equivalent. Multicompartment modeling has proven to yield predictions which are as accurate as those made by physiological models, and the data required can be derived from measurements of tracer/tracee ratios of stable isotopes. Multicompartment models differ in how complex they are. Practically, their design is usually a compromise for what is practically feasible. Too simplified models may not adequately describe the kinetic heterogeneity present within the system. On the other hand, it's hard to generate experimental data for overly complex models.

**Figure 2:**
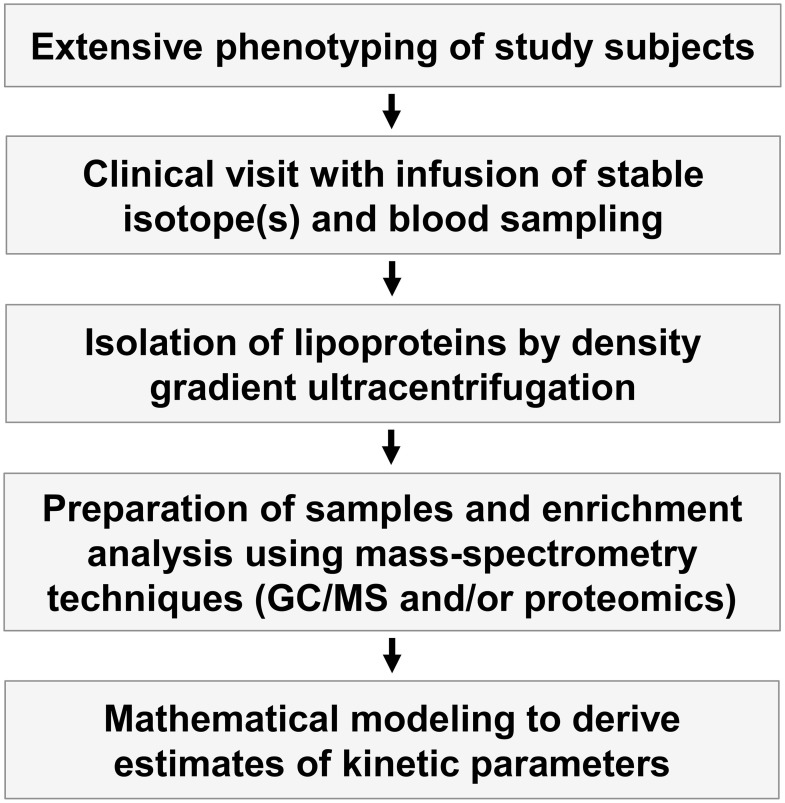
**Outline of kinetic tracer studies**. *In vivo* kinetic studies are complex and involve both extensive phenotyping of study subjects, clinical studies where stable isotopes are infused into the study subjects and blood samples are taken, isolation of lipoprotein fractions by ultracentrifugation, enrichment studies of the stable isotopes using different mass spectroscopy techniques, and mathematical modeling of the enrichment curves to derive indices of kinetic variables.

The power of mathematical modeling to describe the metabolic pathways of lipid and lipoprotein metabolism was first demonstrated by Drs. Berman and Zech (Grundy et al., 1979; Zech et al., 1979). Since then, kinetic studies combined with mathematical modeling have been used to clarify the pathogenesis of impaired lipoprotein metabolism in humans linked to accelerated CVD, obesity, and insulin resistance (Figure 2). The methodology has also been instrumental in testing how efficiently novel drugs improve the dyslipidemia. However, it's important to emphasize that all models are based on several assumptions and simplifications. Therefore, mathematical modeling does not determine the kinetics of lipids directly; rather, they derive an indirect approximation.

## Models to study VLDL kinetics

Increased levels of apoB-containing lipoproteins are the most important risk-factor for developing CVD. It's therefore clinically relevant to decipher the pathophysiology of impaired metabolism of apoB-containing lipoproteins. Until today most studies have used steady-state models to elucidate VLDL kinetics in the fasting state. However, it can be argued that these studies are not fully physiologically designed since human are in a postprandial state most of the time awake.

To study postprandial lipid metabolism it is necessary to include also intestinal derived lipoproteins. The majority of studies of intestinal lipid metabolism has been conducted in a “constant feeding” regime where small meals are served frequently to achieve a steady state of the intestinal lipoprotein secretion (Lamon-Fava et al., 2007; Sun et al., 2013; Padilla et al., 2014; Tremblay et al., 2014; Xiao et al., 2014). To date, only a few studies have focused on apoB48 metabolism following a single meal (Wong et al., 2014a,b). No studies have yet linked hepatic and intestinal lipid metabolism in a combined model, and therefore it is not possible to study the interaction of these lipoproteins as depicted in Figure 1. From fat load studies it is obvious that there is a clear interplay between hepatic and intestinal lipoproteins (Adiels et al., 2012). Therefore, such models must include that apoB48- and apoB100-containing particles are cleared from the circulation by common pathways and therefore compete for clearance (Brunzell et al., 1973). During the postprandial phase, other factors need also to be considered such as insulin secretion which may affect hepatic lipoprotein secretion (Lewis et al., 1993; Adiels et al., 2007; Sorensen et al., 2011; Sondergaard et al., 2012).

In steady-state modeling of VLDL kinetics it has been discussed whether VLDL-apoB and VLDL-TG should be modeled in the same integrated model, or if they should be modeled independently. In integrated models the equation for the rate of change of an apoB100 compartment is linked to the rate of change of the corresponding triglyceride compartment size. This procedure of tying together the apoB100 and triglyceride models enhances the precision of the model as a whole. As each particle contains one single copy of apoB100, the model provides an estimate of the lipolytic rates (the loss of triglycerides per time unit), which can then be used as a physiological readout for answering study questions related to dyslipidemia. Drs. Ramakrishnan and Ginsberg recently reported that the VLDL-apoB and VLDL-TG pools in the delipidation cascade have identical rate constants despite different fates and mass distribution (Ramakrishnan and Ginsberg, 2015). These results strongly support integrated steady-state models of VLDL-apoB and VLDL-TG kinetics.

**Figure 3:**
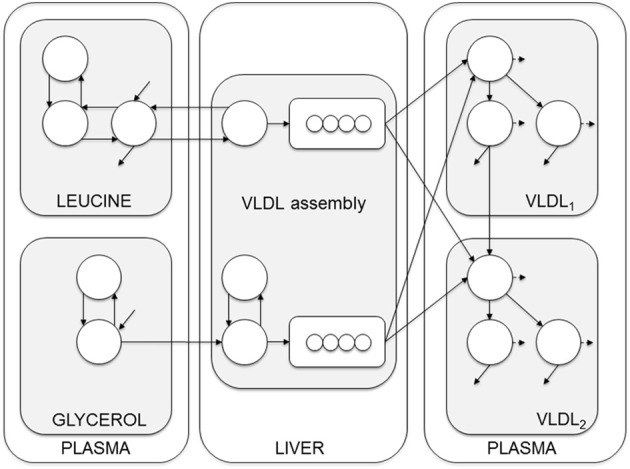
**Compartment model of VLDL apoB and triglyceride kinetics**. The model includes separate modules for leucine and glycerol. Plasma leucine kinetics is modeled using a four-compartment system that drives the synthesis and secretion of apoB into VLDL_1_ and VLDL_2_. Plasma glycerol kinetics is modeled using a two-compartment system connected to fast and slow pathways for triglyceride (TG) synthesis. Plasma apoB and triglyceride kinetics are modeled using a four-compartment hydrolysis chain, in which the kinetics of apoB and triglyceride coupled. For each apoB compartment, there is an equivalent compartment for triglyceride. Triglycerides hydrolyzed from VLDL particles are represented by the dashed arrows, and particles lost from the plasma space are represented by the solid arrows. See Adiels et al. (2005) for additional model details.

The major predictor of plasma triglycerides are VLDL_1_-TG. We therefore developed a multicompartment model that allows the kinetics of triglycerides and apoB100 in VLDL_1_ and VLDL_2_ to be simultaneously assessed after a bolus injection of glycerol and leucine stable isotopes (Figure 3) (Adiels et al., 2005). Analysis of tracer/trace curves of the stable isotopes in VLDL_1_ and VLDL_2_ was used to derive estimates of kinetic parameters using mathematical modeling. By integrating apoB and triglycerides in the model, the triglyceride: apoB ratio of newly produced VLDL_1_ and VLDL_2_ particles can be estimated to follow the transfer and removal of lipids (Adiels et al., 2005).

The model can be envisioned as a two-layer model, connected at certain points and is based on the apoB model as described by Packard et al. (1995). The model consists of four parts; plasma leucine, plasma glycerol, the assembly of lipoprotein, and lipoprotein plasma kinetics. The plasma kinetics is modeled by a four-compartment hydrolysis chain, where the apoB and triglyceride kinetics are coupled at the transfer between compartments. Removal from triglyceride compartments consists of both removal of whole particles and removal of triglycerides.

## Individual and population kinetics

Traditionally, parameters for each individual are estimated individually and conclusions are made based on some statistical model applied to the model output. The model complexity ranges from one single compartment models describing VLDL-TG-kinetics (Patterson et al., 2002), to 12 compartments describing the combined apoB and TG kinetics in VLDL1 and VLDL2 (Adiels et al., 2005). The majority of published studies have used the SAAMII software (The Epsilon Group, US) (Barrett et al., 1998).

As models are becoming more complex and includes more unknown parameters, more data is needed to support the estimation of the model parameters. Individual data sets are also sensitive to loss of data and data quality, which is directly reflected in the variability in the estimated parameters.

Modern modeling techniques combine the mechanistic model (describing the system) with a statistical model (describing the populations). This is an extension to non-linear mixed effects models (NLME) where the non-linear model is the set of ordinary differential equations describing the system. Model parameters are described as random variables drawn from a distribution centered round the population mean. Using data from all individuals, population means and variances as well as individual estimates are calculated for all parameters (Beal and Sheiner, 1982).

The major gain using NLME approaches are that generally the estimated variances are smaller compared to the traditional approach, thus statistical power is greatly increased. Furthermore, such methods have shown produce better results also when data are sparse (Denti et al., 2009; Largajolli et al., 2012). Using these techniques we have recently shown that estimation of lipoprotein kinetics parameters can greatly be improved by an NLME approach (Berglund et al., 2012) (leucine subsystem) and (Berglund et al., 2015) (full system).

To estimate the day-to-day variability in VLDL kinetics and other measures, Magkos et al. repeated a kinetic study in 8 obese men on two occasions 2 months apart (Magkos et al., 2011). Using this data they calculated the sample size needed to detect differences (15–35%) between two groups during an intervention. For VLDL-TG secretion, *n*=15 was needed in each group to detect a difference of 25% using an unpaired study design at a study power of 80%. We compared 15 healthy controls and 15 type 2 diabetic subjects and found that using the traditional approach, *n*=9 was needed to detect the difference in VLDL_1_ secretion. In contrast, using the NLME approach, a sample size of only *n*=5 was needed to detect the same difference (Berglund et al., 2015).

The limitations for these methods are so far that they are very computational intensive and the methods are not yet implemented in traditional software.

## Pathophysiology of dyslipipidemia in obese subjects

**Figure 4:**
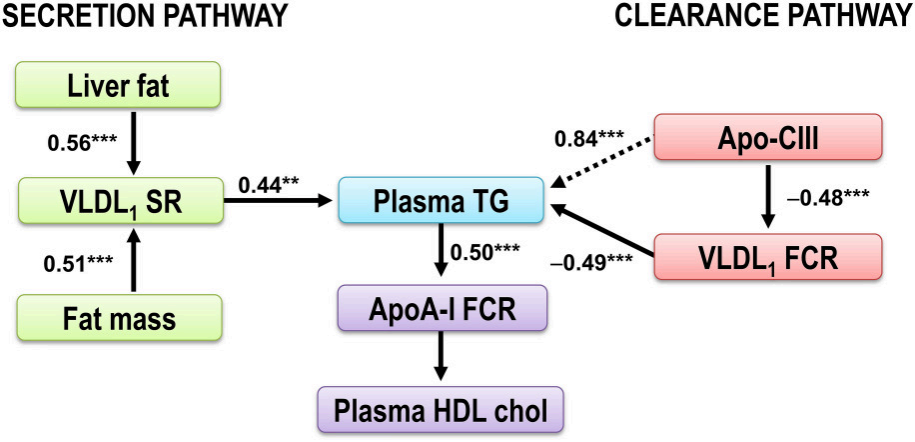
**The dysregulation of the VLDL_1_-TG and apoAI metabolism in obese subjects**. The plasma triglycerides are determined by the balance between synthesis and removal of VLDL_1_-TG, illustrated by a *Synthesis pathway* and a *Clearance pathway*. The *Synthesis pathway* includes liver fat and total fat mass, the two independent predictors of VLDL_1_–triglyceride secretion rate. In the *Clearance pathway*, plasma concentration of the LPL inhibitor apoC-III was tightly linked with plasma TG and catabolism of VLDL_1_-TG. In addition, apo-CIII was closely related to plasma TG concentration (dotted arrow). This indirect effect of apoC-III on plasma triglycerides is likely explained by effect(s) of apoC-III beyond LPL-independent pathways of triglyceride metabolism (Huard et al., 2005; Sacks et al., 2011). Increased plasma triglycerides are associated with increased HDL catabolism (Pont et al., 2002; Ooi et al., 2005; Chan et al., 2009). Thus, increased plasma triglycerides are closely associated with decreased HDL cholest erol (Verges et al., 2014). Figure modified from Borén et al. (2015). ^**^p < 0.01; ^***^p < 0.001.

To elucidate the pathophysiology of the dyslipidemia in subjects with abdominal obesity, we recently performed lipoprotein kinetic studies in 46 middle-aged well-phenotyped men and women with abdominal obesity and additional cardiometabolic risk factors to clarify determinants of plasma triglyceride concentration (Borén et al., 2015). The results are summarized in Figure 4. The concentration of triglycerides in plasma is determined by the balance between synthesis and removal of VLDL_1_-TG. Thus, dual metabolic defects are required to produce hypertriglyceridemia in obese subjects (Taskinen et al., 2011). To illustrate this, two pathways are shown; a *Synthesis pathway* and a *Clearance pathway*. The *Synthesis pathway* include liver fat and total fat mass since these remained independent predictors of VLDL_1_–triglyceride secretion rate in a stepwise multivariable regression analysis. In the *Clearance pathway*, plasma concentration of the LPL inhibitor apoC-III was tightly linked with plasma TG and catabolism of VLDL_1_-TG. In addition, apo-CIII was closely related to plasma TG concentration. This indirect effect of apoC-III on plasma triglycerides is likely explained by effect(s) of apoC-III beyond LPL-independent pathways of triglyceride metabolism (Huard et al., 2005; Sacks et al., 2011).

HDL cholesterol is closely associated with diabetic dyslipidemia and abdominal obesity (Verges et al., 2014), and low HDL cholesterol is strongly associated with increased cardiovascular risk (Ninomiya et al., 2004). Pharma industry has therefore developed drugs that increase HDL cholesterol. However, clinical studies with these agents have not been successful, and the causative role of HDL is therefore questioned. This indicates that low HDL cholesterol is more a marker of an atherogenic lipoprotein profile. *In vivo* kinetic studies performed in abdominally obese individuals have shown that low plasma concentration of HDL cholesterol is the consequence of increased HDL catabolism (Pont et al., 2002; Ooi et al., 2005; Chan et al., 2009), and increased plasma triglycerides is closely associated with decreased HDL cholesterol (Verges et al., 2014).

Interestingly, when comparing the *Synthesis pathway* and the *Clearance pathway*, indices of catabolism were stronger predictors of plasma triglycerides than parameters of secretion. In a multivariable regression model, VLDL_1_-TG kinetics explained 76% of the variation in the total plasma triglycerides. Kinetic parameters of VLDL_1_-TG secretion explained 19–20% only of the variation in plasma triglyceride concentrations in the study subjects. Thus, in subjects with abdominal obesity and dyslipidemia, the VLDL_1_-triglyceride clearance is a stronger determinant of the plasma triglyceride concentration than increased secretion of VLDL_1_ particles. This finding may support combination therapies in subjects with abdominal obesity and dyslipidemia affecting both secretion and catabolism of VLDL_1_-TG. The results further support apoC-III as a key target for reducing residual cardiovascular risk.

## Liver fat accumulation and CVD

The observed associations between liver fat plasma triglycerides are in line with earlier studies showing that liver fat content is closely related to triglyceride secretion in different settings and populations (Chan et al., 2010; Taskinen et al., 2011) and a better predictor of triglyceride secretion than intra-abdominal fat (Fabbrini et al., 2009; Magkos et al., 2010). It is also clinically important because of the worrisome increase of non-alcoholic fatty liver disease (NAFLD), defined as hepatic fat accumulation that exceeds 5% of liver weight in individuals who do not consume significant amounts of alcohol (Neuschwander-Tetri and Caldwell, 2003; Vernon et al., 2011). Approximately 25% of adults have NAFLD, and its prevalence increases to 70–90% among adults with obesity or type 2 diabetes (Ray, 2013). Even though NAFLD may progress to severe liver diseases, the most common cause of death in patients with NAFLD is CVD. Several epidemiological studies indicate that NAFLD is not merely a marker of CVD, but may also be actively involved in its pathogenesis (Targher et al., 2008). Thus, the finding that NAFLD is closely linked to overproduction to of VLDL_1_-TG that drives an atherogenic dyslipidemia characterized with hypertriglyceridemia, HDL-cholesterol, and postprandial hyperlipidemia provide an important explanation for this and indicates that novel treatments reducing liver fat accumulation might be important in preventing CVD (Boren et al., 2013).

## Conclusion

Lipid homeostasis is essential for human health but elevated lipid levels are a risk factor for atherosclerosis and thus can lead to symptomatic CVD. Increased lipid levels can be caused by either increased secretion of atherogenic lipoproteins, and/or impaired clearance of lipoproteins from the circulation. The use of *in vivo* kinetic studies using stable isotopes and mass spectrometry in combination with the development of mathematical models has been critical in advancing understanding of normal and dysregulated lipid metabolism. However, kinetic studies are time-consuming, expensive and require a high level of expertise. Thus, they are limited to rather few research groups. Future, development will hopefully enable us to optimize the protocols and increase the statistical power, in particular when data are sparse (Denti et al., 2009; Largajolli et al., 2012).

Also, combining kinetic studies with advanced modeling, as genome-scale metabolic modeling (Mardinoglu and Nielsen, 2015; O'Brien et al., 2015), might provide even deeper understanding of the metabolic changes and clarify the underlying metabolic perturbations in the occurrence of metabolism related disorders. Genome-scale metabolic models (GEMs) are the collection of the biochemical reactions and associated protein-coding genes, and provide a scaffold for integration of the fluxomics as well as other omics data e.g., proteomics, transcriptomics, metabolomics, and lipidomics. Recently, a functional GEM for the hepatocytes in liver tissue has been reconstructed (Mardinoglu et al., 2014) and its use together with the kinetic studies may provide detailed knowledge for understanding the relationship between the genotype-phenotype in different clinical conditions.

### Conflict of interest statement

The authors declare that the research was conducted in the absence of any commercial or financial relationships that could be construed as a potential conflict of interest.
